# Evidence of weight loss in junior female judo athletes affects their development

**DOI:** 10.3389/fspor.2024.1420856

**Published:** 2024-06-13

**Authors:** Ena Yoshida, Harumi Hayashida, Tomonobu Sakurai, Kenzo Kawasaki

**Affiliations:** Graduate School of Sports Sciences, Toin University of Yokohama, Yokohama, Japan

**Keywords:** adolescence, female athlete, growth spurt, height, menstruation

## Abstract

**Purpose:**

The facile manipulation of body weight in junior athletes has the potential to pose significant risks to their lifelong health. In judo, which is a weight class sport, pre-competition weight loss is widespread even among juniors, but information on the current situation is scarce, especially for female athletes, for whom it is important to provide adequate nutrition and enhance bone mass during the growth period, and the details of the current situation are not clear. Therefore, the purpose of this study was to determine the actual weight loss during the growth period in junior female judo athletes and its subsequent impact on their health.

**Methods:**

The survey was a cross-sectional survey of junior female judo athletes in Japan using a questionnaire. Participants were asked to respond via an online questionnaire about their weight, height, weight loss experience, menstruation, competition results, and other lifestyle.

**Results:**

51.8% of subjects experienced weight loss for competition during their junior high school years (ages 12–15). Those who experienced weight loss during secondary sexual characteristics were found to be significantly shorter in current height than those who did not (*p* < 0.05). Weight loss during secondary sexual characteristics did not affect current menstrual cycle. There was no significant difference in competition results due to the experience of weight loss during junior high school (*χ*^2^ = 4.485, df = 3, n.s.).

**Conclusions:**

These findings suggest that weight loss during the growth spurt phase may adversely impact normal development. It also suggested that weight loss during the junior high school years may not be a strategy to bring about better competition results. These observations indicate the need for education on appropriate class selection and weight control for junior athletes in weight class competitions.

## 1 Introduction

In judo, since the competition performance is likely to depend on the body weight (1), a weight class system is adopted to avoid such inequality, and athletes are required to weigh in and clear a delimited weight range before a match. It is known that the majority of senior judo athletes prefer to compete in a lower weight class than their normal weight and adjust their weight prior to competition by weight loss using gradual or rapid methods (2, 3). Gradual weight loss is achieved by dropping body fat by creating a negative energy balance through increased physical activity and decreased energy intake from food. Rapid weight loss is a weight loss technique in which water and carbohydrates are severely restricted or consumed, and extremely short-term, drastic weight loss is implemented in an attempt to temporarily clear the prescribed weight only at the weigh-in. In particular, rapid weight loss is of concern in terms of its safety aspects, psychological (4–6), decreased immune capacity (7), increased muscle damage (8), and impaired performance (9, 10). Despite all the health risks, rapid weight loss is very prevalent because of the potential for competitive advantage in cases of adequate recovery from post-weigh-in to competition (11). Furthermore, numerous athletes find themselves compelled to rapid lose weight in order to avoid being at a competitive disadvantage (12).

On the other hand, judo is now a sport that is popular among people of all ages (13), and international competitions for juniors are held every year. In most cases, weight limits are set for junior competitions as well, and, like senior judo athletes, they must weigh in and clear the weight limits prior to competition. Berkovich et al. reported that many (74%) of junior athletes experienced rapid weight loss before the age of 13 (14), indicating that even juniors practice weight adjustment for competition as a common practice it can be seen that. In addition, since losing a moderate amount of body fat in preparation for competition improves instantaneous force and other aspects of judo performance (15), weight adjustment is a fundamental aspect of athleticism, but junior athletes differ greatly from senior athletes in both mental and physical aspects. Weight loss (pressure to lose weight), such as weight manipulation and food restriction, is a risk factor for eating disorders (16, 17) and is also known to increase the risk of injury occurrence (18) Normally, children are at a time when nutritional accumulation is important for tissue growth. Even if it is gradual weight loss, going through weight loss with an immature body and mind is not only a transitory increase in burden, but also a risk to lifelong health.

Specific discussions and actions have been slow to address this widespread manipulation of junior athletes' body weight to compete. This may be due to the lack of research to elucidate the circumstances surrounding junior judo athletes and the accumulation of scientific evidence for the effects of subsequent health conditions. Little is known about the current status of weight loss and its effects specifically on female junior athletes (19). For female athletes, adequate hormone production during the growth period is important for the acquisition of sufficient bone mass and subsequent reduction of health risks (20). And the higher incidence of eating disorders compared to males should also be fully noted (21–23).

Therefore, this study was conducted with the aim of understanding the status of weight loss among Japanese junior female judo athletes during their developmental stages (elementary school, junior high school, and high school), and to clarify their past weight loss experiences and the effects of weight loss on their current development.

## 2 Material and methods

### 2.1 Research design

This survey was a cross-sectional study using an online questionnaire.

### 2.2 Subjects

In Japan, a system of school-based club activities has been established since around 1,880, and it was assumed that most athletes registered with the All Japan Judo Federation belonged to judo clubs in schools in Japan. Therefore, the target population was high school female judo players who belonged to judo clubs in high schools in Japan. Requests for participation in the questionnaire were extended to schools at all levels of competition, ranging from beginners to participants in national tournaments. The Japanese grade and age divisions are 6–12 for elementary school students, 12–15 for junior high school students, and 15–18 for high school students.

### 2.3 Survey period

The questionnaire was operational from January 6 to February 28, 2023.

### 2.4 Questionnaire request and methodology

The survey was conducted in an online format (Google Forms: Google LLC). Letters were mailed to 417 schools in Japan that were thought to have high school female judo players based on tournament lists of high school judo tournaments held in Japan over the past two years that were available on the Internet and on each school's website. The letters consisted of a request letter and a printed QR-coded survey link. After mailing the letters to each school, we asked each prefectural judo federation to disseminate the questionnaire and promote its collection.

### 2.5 Questionnaire contents

The survey encompassed inquiries regarding current habits and past experiences. The questionnaire consisted of (1) Basic information: grade, height, weight, weight class, results of elementary, junior high, and high school (Top 4 or more in the national competition, Participation in the national competition, Participation in the regional tournament, and Results not meeting the above criteria) (2) Current eating habits (3) Weight management (4) Sleeping time (5) Practice time (6) Past weight loss experience (7) Menstruation-related (age at menarche, current menstrual cycle, and menstrual symptoms).

All questions were administered in a recall method that relied on the respondent's memory. The questionnaire was developed with reference to several content areas. Specific content and considerations are listed next. Basic information was obtained from previous studies (24, 25). Competition results were combined for both individual and team competitions, and subjects were asked to select the best competition results. The Dietary Variety Square (DVS) (26, 27) developed by Kumagai was used to assess current eating habits. Subjects were asked to indicate their weekly frequency of consumption of 10 food groups: meat, seafood, eggs, milk, soy products, green and yellow vegetables, seaweed, fruits, potatoes, and fats and oils. The total score represents the food intake diversity, with “eat almost every day,” received 1 point and “eat once every 2 days,” “eat once or twice a week,” and “rarely eat” received 0 point. The distribution of scores is from 0 to 10. The higher the score, the better the diversity of food intake. Weight management was created by referring to a previous study (25) for the options regarding information on consultants. Responses were made in a multiple-response format. Sleep time was taken from the Pittsburgh Sleep Quality Index (PSQI) up to question 4, which was differentiated from bedtime, and respondents were asked to specify the time they were actually asleep. The practice time question was asked with reference to previous studies (24, 25), distinguishing between weekdays and non-school days. “Weight loss” was defined as “intentional weight loss for the purpose of competing in a tournament, occurring within the period from one month before to the day of the competition.” This definition included cases where the weight loss process started just a few days before the competition. And respondents were asked to indicate whether they had or had not lost weight in each of the elementary, junior high, and high school categories. Menstruation-related data were collected to examine the proportion of amenorrhea. Respondents were asked whether they had normal menstruation (approximately every month) or no menstruation. If they had no menstruation, they were presented with options to classify it as oligomenorrhea (irregular but within 3 months), secondary amenorrhea (irregular but between 3 and 6 months, or irregular and absent for more than 6 months), or Amenorrhea (no menstruation yet). Menstrual symptoms were evaluated using the Menstrual Distress Questionnaire (MDQ), consisting of 47 questions concerning physical and emotional changes associated with the menstrual cycle, in which participants report the degree of their symptoms on a scale of 0, 1, 2, or 3 per question. Higher scores indicate more intense symptoms. In this survey, respondents were asked to focus on symptoms during menstruation.

### 2.6 Questionnaire and analysis procedures

**Figure 1:**
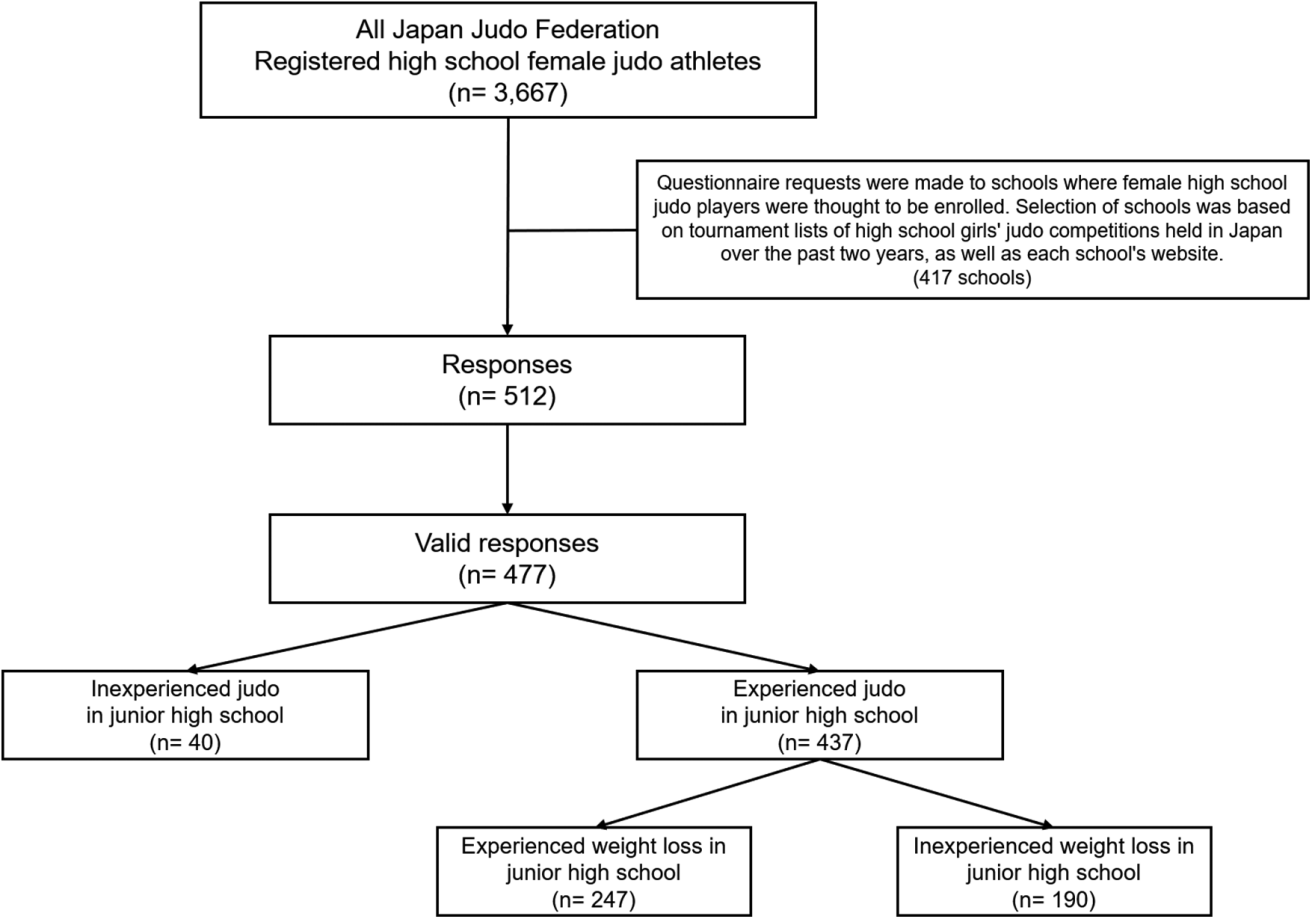
Questionnaire and analysis procedure.

Figure 1 illustrated the overall grouping at the time of questionnaire administration and analysis. Focusing on the junior high school years, when the majority of the participants had experienced weight loss, the participants were divided into two groups: those who had experienced weight loss in junior high (*n* = 247) and those who had not experienced weight loss (*n* = 190). The current height, competitions result at that time, and current menstrual status were then compared.

### 2.7 Ethical consideration

Participation in this study was voluntary for the subjects. The printed text of the survey QR code and the start page of the online survey explained the purpose, content, and methods of the study, how the data will be stored, and that the data will be compiled, analyzed, and published in such a way that individuals cannot be identified. Participants voluntarily indicated their consent to participate and agreed to have their data analyzed. This study was conducted with the approval of the Toin University of Yokohama Ethics Committee (Approval No. I-66).

### 2.8 Statistical analysis

The obtained data were presented as mean ± standard deviation or value (percentage). The normality of the data was assessed using the Kolmogorov-Smirnov test. An unrepeated two-way analysis of variance (group × weight class) was conducted for height, where normality was observed, and a *t*-test was performed for MDQ score. For the diversity of food intake, where normality was not observed, the Mann-Whitney *U* test was employed to examine differences in score distribution, and the Kruskal-Wallis test was used to analyze differences in age at menarche between groups. The chi-square test was utilized to compare proportions between groups regarding weight loss experience in junior high school in relation to competition results and current menstrual cycle status. Relationships between variables were examined using Pearson's correlation analysis. Statistical processing was performed using SPSS ver. 29.0.1.1 (IBM Corporation). The respective statistical significance level was set at less than 5%.

## 3 Results

### 3.1 Valid responses

The total number of responses obtained for this study was 512, of which 477 were valid responses (valid response rate: 93.16%).

### 3.2 Subjects attributes and characteristics

The total number of respondents was 211 high school 1st-year students (44.2%), 193 high school 2nd-year students (40.6%), and 73 3rd-year students (15.3%). The distribution of the subjects' weight classes (i.e., the classes they will compete in the next competition) was as follows: 48 kg class: 82 (17.2%), 52 kg class: 95 (19.9%), 57 kg class: 109 (22.6%), 63 kg class: 79 (16.6%), 70 kg class: 48 (10.1%), 78 kg class: 33 (6.9%), 78+kg weight class: 31 (6.5%). The current weight of all (*n* = 477) was 60.5 ± 12.2 kg Current height was 158.9 ± 5.6 cm.

### 3.3 Weight loss experience

**Figure 2:**
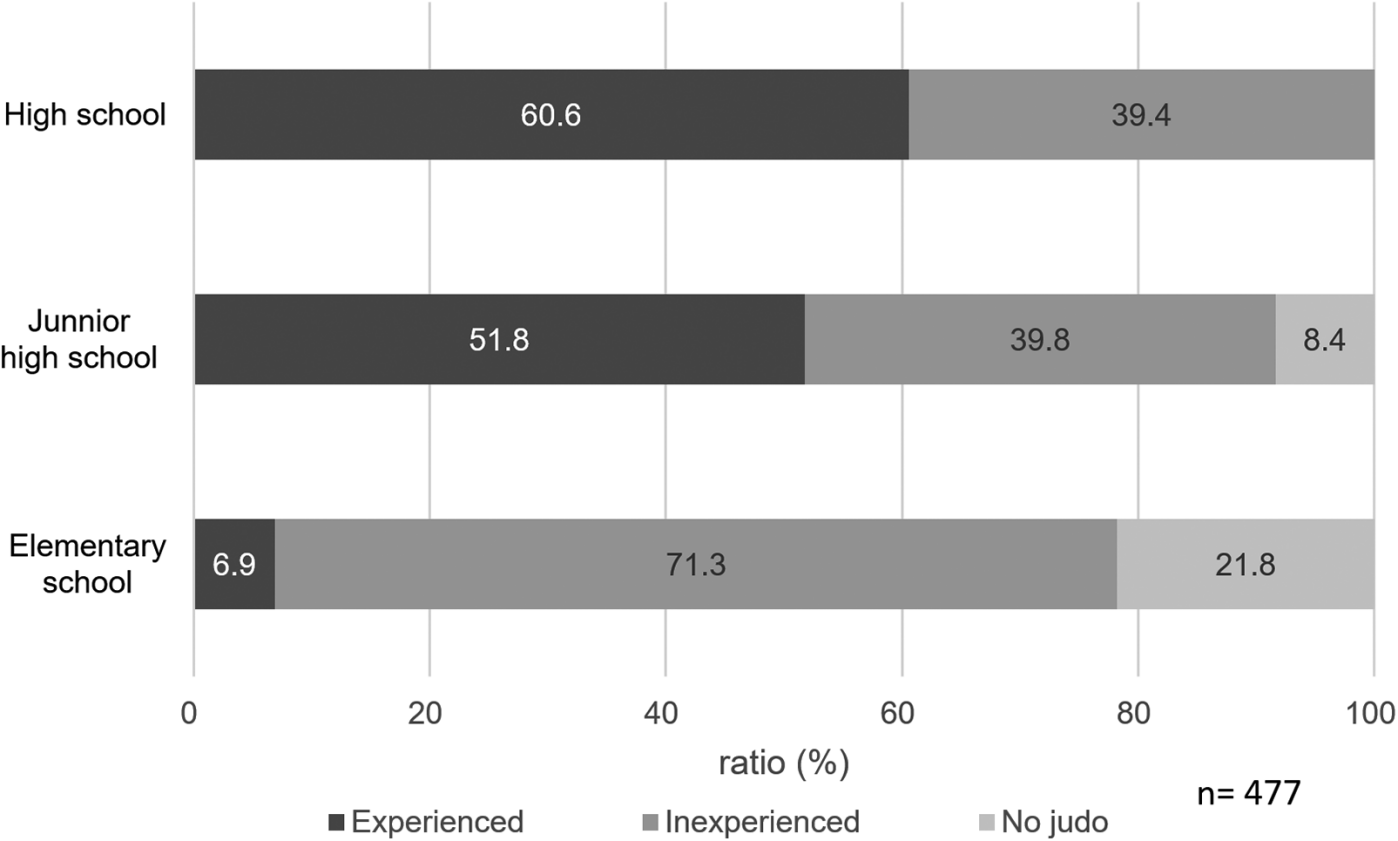
Percentage of elementary, junior high, and high school students who have experienced weight loss. “Experienced” indicates those who experienced weight loss, “Inexperience” indicates those who did not experience weight loss, and “No judo” indicates that they did not experience judo at the time.

Figure 2 showed the percentage of subjects who had experienced weight loss in the past. More than 50% of the subjects had experienced weight loss as junior high school students.

### 3.4 Relationship between weight loss in junior high school and height

**Figure 3:**
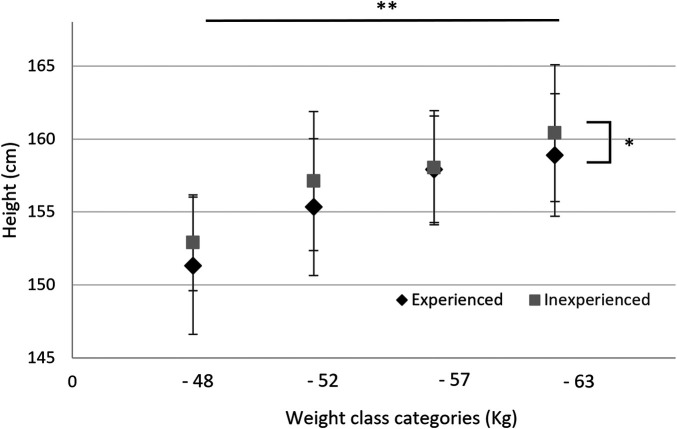
Difference between with and without weight loss in junior high school and current height.

A comparison of current height based on the presence or absence of weight loss during junior high school revealed significant differences in height based on the factor of presence or absence of weight loss among subjects in the 48 kg, 52 kg, 57 kg, and 63 kg weight classes, with the group that experienced weight loss during junior high school being significantly shorter than the group that did not (Figure 3). No such differences were found in the 70 kg, 78 kg, and 78 kg + weight classes. Significant differences in height were found among the seven weight classes (*p* < 0.01), and the heavier the weight class, the taller the average height tended to be.

### 3.5 Effect of junior high school weight loss experience on competition result

A comparison of the competition results at that time between the group that experienced weight loss in junior high school and the group that did not show any significant difference (*χ*^2^ = 4.485, df = 3, n. s.) (Table 1).

**Table 1:** Weight loss experience in junior high school and competition results at that time.

	Experienced weight loss group *n* (%)		Inexperienced weight loss group *n* (%)	
Top 4 or more in the national competition	17	(6.9)	12	(6.3)
Participation in the national competition	64	(25.9)	51	(26.8)
Participation in the regional competition	75	(30.4)	42	(22.1)
Results not meeting the criteria above	91	(36.8)	85	(40.3)
Total number	247		190	

### 3.6 Age at menarche, current menstrual cycle and associated menstrual symptoms

The age at menarche for all subjects was 12.21 ± 1.45 years (*n* = 475), with the age at menarche for each group detailed in Table 2. The current menstrual cycle distribution among all subjects was as follows: approximately 1 month for 338 (70.9%), irregular but within 3 months for 114 (23.9%), irregular but between 3 and 6 months for 19 (4.0%), irregular and absence for more than 6 months for 4 (0.8%), and not yet menstruated was reported by 2 subjects (0.4%). Comparisons were made between groups based on whether they had experienced weight loss during junior high school within the categories “approximately 1 month”, “irregular but within 3 months”, and “irregular but between 3 and 6 months”, where comparisons were possible and no significant differences were found (*χ*^2^ = 0.700, df = 2, n.s.). Due to the small sample sizes, the categories “irregular and absence for more than 6 months” and “not yet menstruated” were not included in the analysis.

**Table 2:** Information on the age at menarche of the subjects.

Weight class categories	All	−48 kg	−52 kg	−57 kg	−63 kg	−70 kg	−78 kg	78 + kg	*P* value
All subjects	12.21 ± 1.45 (*n* = 475)	12.57 ± 1.57 (*n* = 81)	12.47 ± 1.46 (*n* = 95)	12.29 ± 1.34 (*n* = 108)	12.15 ± 1.47 (*n* = 79)	12.00 ± 1.35 (*n* = 48)	11.42 ± 1.10 (*n* = 33)	11.52 ± 1.34 (*n* = 32)	**
No judo	12.00 ± 1.45 (*n* = 40)	12.00 ± 1.61 (*n* = 10)	12.56 ± 1.83 (*n* = 9)	11.92 ± 1.07 (*n* = 13)	11.67 ± 1.25 (*n* = 3)	11.75 ± 0.43 (*n* = 4)	0.00 ± 0.00 (*n* = 0)	10.00 ± 0.00 (*n* = 1)	–
Played Judo	12.23 ± 1.45 (*n* = 435)	12.65 ± 1.55 (*n* = 71)	12.47 ± 1.41 (*n* = 86)	12.34 ± 1.37 (*n* = 95)	12.17 ± 1.47 (*n* = 76)	12.02 ± 1.41 (*n* = 69)	11.42 ± 1.10 (*n* = 33)	11.57 ± 1.33 (*n* = 30)	**
Experienced weight loss	12.39 ± 1.41 (*n* = 246)	12.63 ± 1.54 (*n* = 48)	12.52 ± 1.44 (*n* = 61)	12.32 ± 1.33 (*n* = 59)	12.46 ± 1.40 (*n* = 41)	12.38 ± 1.27 (*n* = 16)	11.40 ± 0.80 (*n* = 15)	11.83 ± 0.90 (*n* = 6)	n. s.
Inexperienced weight loss	12.02 ± 1.49 (*n* = 189)	12.70 ± 1.57 (*n* = 23)	12.32 ± 1.32 (*n* = 25)	12.36 ± 1.42 (*n* = 36)	11.83 ± 1.48 (*n* = 28)	11.82 ± 1.44 (*n* = 28)	11.40 ± 0.80 (*n* = 18)	11.50 ± 1.41 (*n* = 24)	*

“No judo” and “Played judo” based on judo experience in junior high school.

* *p* < 0.05, **p < 0.01, n. s.: not significant.

Comparison of MDQ scores showed no difference based on weight loss experience in junior high school, and menstrual MDQ scores were only significantly correlated with sleep duration in the past month (*r* = 0.10, *p* = 0.04, *n* = 475).

### 3.7 Lifestyle of the subjects

The subjects current practice time on weekdays was 168 ± 47 min, and on non-school days 212 ± 74 min. The average sleep duration over the past month was 396 ± 58 min.

The diversity score of current food intake, where a higher score indicates a better diversity of food intake, was found to be significantly lower in the group that experienced weight loss in junior high school compared to the group that did not experience (3.7 ± 2.6 vs. 4.3 ± 2.7 point, *p* = 0.04) (Table 3).

**Table 3:** Comparison of the distribution of diversity scores of current food intake in the experienced and inexperienced weight loss groups.

Score	0	1	2	3	4	5	6	7	8	9	10	Mean ± SD
Experienced weight loss group (*n* = 247)	10.9	13.0	13.8	11.7	13.8	12.6	9.7	4.0	5.3	1.2	4.0	3.7 ± 2.6 point
Inexperienced weight loss group (*n* = 190)	6.8	7.9	11.6	16.3	16.8	11.6	8.9	5.8	3.2	4.7	6.3	4.3 ± 2.7 point

The numbers in the table corresponding to scores 0–10 in the SCORE are percentages (%). Based on Mann-Whitney *U*-test.

### 3.8 Weight management consultants

**Figure 4:**
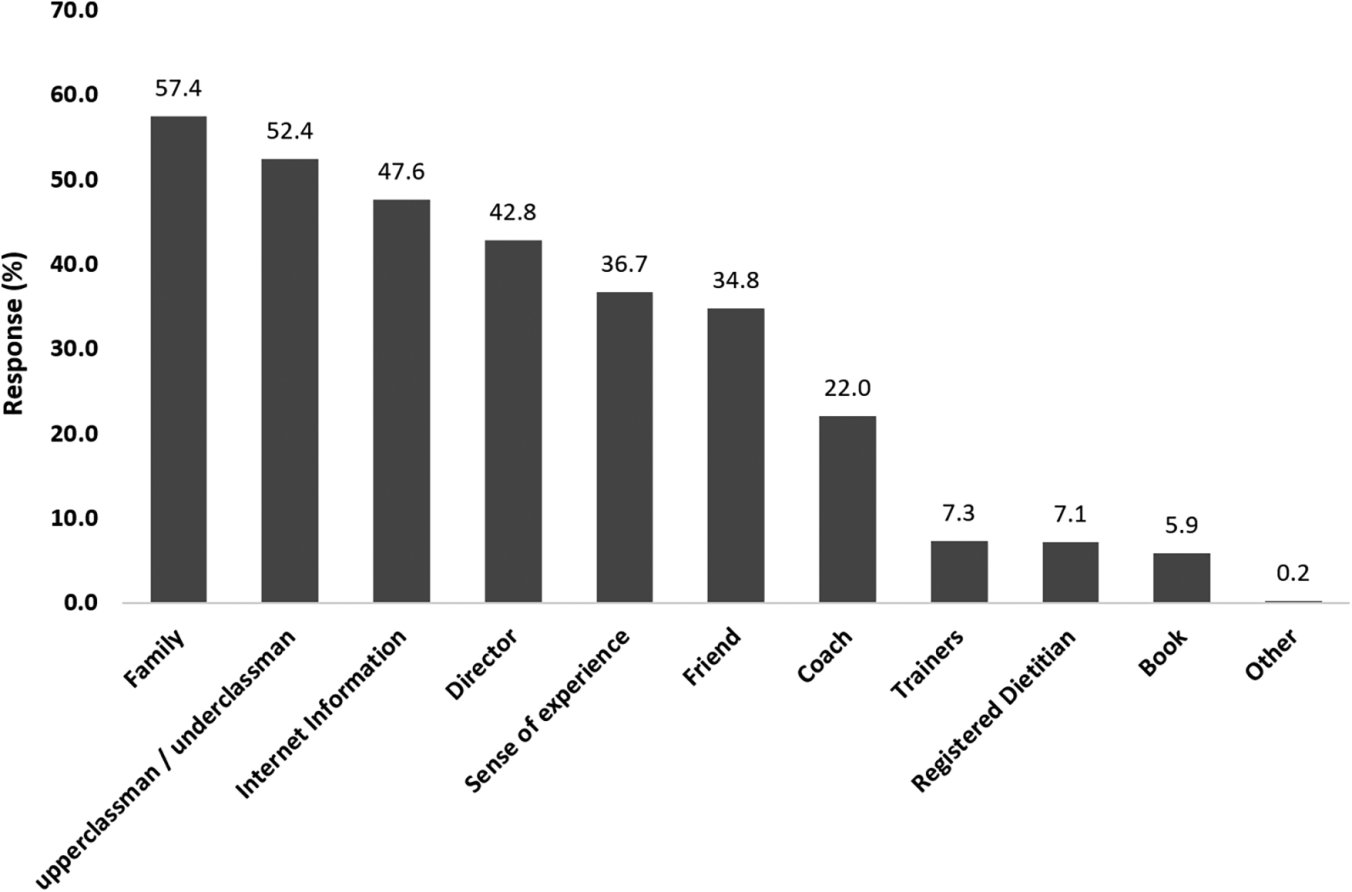
High school female judo athletes current weight advisors. Multiple responses were allowed.

Figure 4 showed the sources of information on eating habits and weight management methods. The most common source was family (57.4%).

## 4 Discussion

The aim of this study was to ascertain the weight loss status of Japanese junior female judo athletes during their developmental stages (elementary school, junior high school, and high school), and to elucidate the effects of weight loss on their development. The survey results revealed that the majority of first- through third-year high school students who participated in the study had experience weight loss during their junior high school years. Furthermore, the findings suggested that weight loss during junior high school, a period characterized by the onset of growth spurts and critical nutritional requirements, may adversely affect bone development. This represents a novel discovery, as no previous reports have addressed the reality of weight loss among junior judo athletes in Japan. And it is imperative that appropriate measures be implemented to address these findings.

In aesthetic sports for females, among many other sports, previous studies have demonstrated that intense exercise and lack of energy during adolescence inhibit bone growth and prevent full height gain (28, 29). The present study indicates that a similar phenomenon was observed with weight loss in judo athletes. First, in subjects belonging to the 48 kg, 52 kg, 57 kg, and 63 kg weight classes, which are lightweight to medium weight classes, the current height was significantly shorter in the group that had experienced weight loss compared to the group that had not experienced weight loss in junior high school (Figure 3). In general, weight loss in martial arts is often achieved by maintaining a negative energy balance (2, 30). On the other hand, normal development in humans is achieved by adequate nutritional supplementation (31, 32). In particular, with regard to height growth, it is known that adequate nutrition and accompanying adequate hormone secretion are essential during the period before height growth stops due to complete fusion of the epiphyseal line, roughly around age 15 in females (31). The results of this study suggest that the one-month or shorter weight loss for judo competitions in junior high school, or the awareness and behavior of trying to maintain weight on a daily basis despite the growth period, were factors that inhibited satisfactory bone growth for these females.

In the 70 kg, 78 kg, and 78 kg + weight classes, no height difference was observed between with and without weight loss. These results corroborate our previously stated hypothesis. Examining the age of menarche among respondents in this study by weight class revealed differences in the age of menarche across classes, with heavier weight classes associated with lower ages of menarche (Table 2). In general, women experience a rapid increase in the secretion of female hormones from around 8–9 years of age to 17–18 years of age, the appearance of secondary sexual characteristics, and rapid growth in height, followed by menarche. Menarche occurs 6 months after reaching “maximum height velocity (PHV),” when bone formation proceeds rapidly (33). Participants in this study experienced menarche approximately between the ages of 12 and 13 in the 48 kg, 52 kg, 57 kg, and 63 kg weight classes, while menarche occurred approximately between the ages of 11 and 12 in the 70 kg, 78 kg, and 78 kg + weight classes (Table 2). This difference in age at menarche is considered to represent a difference in the timing of rapid height growth. Among the heavyweight class athletes, most of them had already passed the period of maximum height velocity (PHV) even if they experienced weight loss within the age of 12∼15, which is the age of Japanese junior high school students, and it is possible that this did not have a significant effect on their current height. In addition, heavyweight class athletes generally have more body fat mass compared to lightweight and middleweight athletes (34, 35). Thus, it is possible that weight class athletes have a large amount of body fat reserves and that even if they reduced their food intake for weight loss, they were able to achieve weight loss while ensuring that they had enough nutrients for growth and did not have a significant impact on their growth. While we were unable to examine weight class changes from junior high school to high school in this study, junior high school students belong to the 40 kg, 44 kg, 48 kg, 52 kg, 57 kg, 63 kg, 70 kg, and 70kg + weight classes, while high school students belong to the 48 kg, 52 kg, 57 kg, 63 kg, 70 kg, 78 kg, and 78kg + weight classes. Considering the positive correlation between weight class and height for both junior high school students (34) and high school students, it is thought that the weight class an individual belonged to in junior high school and high school was generally similar, possibly dependent on the skeletal structure they were born with.

This is not to say that weight loss is acceptable for junior athletes based on their individual physique and growth level. The underlying reason for weight loss is to increase the probability of winning a tournament. However, when evaluating their best competition results in junior high school, there was no discernible difference in tournament outcomes between those who underwent weight loss and those who did not (Table 3). The results of this study suggest that the subjects’ weight loss in junior high school may not have been strategic, scientific, and well-considered. In the present study, the menstrual cycle and menstrual symptoms, which are influenced by the nutritional intake, did not differ depending on whether the subjects had experienced weight loss in junior high school or not, suggesting that the intensity of symptoms may be influenced by current lifestyle habits. However, our data revealed a tendency toward delayed onset of menarche among individuals who had participated in judo during junior high school (Table 2). Notably, both women who had not experienced menarche by the time they reached high school were affiliated with the group that had engaged in judo during junior high school. The phenomenon of female athletes exhibiting delayed menarche compared to the general female population has been underscored in numerous studies (36, 37). For female's bodies, strenuous exercise is considered to have no small effect on the maintenance of reproductive development. Adding to this the burden of intentional undernutrition is undesirable. To begin with, it is important that children enjoy sports and compete in a healthy development (38, 39). The way they spend their growing years and their diet have a significant impact on future reproductive function, bone, muscle, and brain development (40–42). Therefore, weight manipulation strategies such as weight loss warrant special attention. Repeated weight loss can cause a decrease in basal metabolism and increase the likelihood of obesity in the future (43–46). And bone formation during school age and young adulthood is crucial for maintaining healthy bones throughout adulthood and beyond (47). Hence, it is very important that strengthening of children with promising futures also be done carefully and appropriately based on their stage of growth.

Note that because the questionnaire was designed to be answered from past memory, we did not use a detailed question focusing only on rapid weight loss, such as a weight loss of 5% or more in one week. The combat sports-specific rapid weight loss techniques that have been noted as generous health risks in senior athletes are probably prevalent in juniors (14), and special effects could also be observed if the number of experiences and actual weight loss ranges were closely examined and investigated.

Suggestions for addressing the current situation were derived from the data of this study. A diverse intake of food groups is typically essential to achieve adequate nutritional balance (48, 49). Scores indicating such food intake diversity were lower in the group that experienced weight loss during junior high school in this study compared to the group that did not undergo weight loss (Table 3). This suggests that the group that experienced weight loss during junior school may have previously lacked opportunities for nutrition education and environmental preparation in meal settings. In addition, it was found that the majority of the subjects would rely on family members and supervision, with very few relying on a nutrition specialist, a dietitian, as an advisor for diet and weight management (Figure 4). Therefore, addressing this issue may necessitate nutrition education aimed at promoting healthy eating habits, as well as education on the concept of appropriate weight class selection and weight control for children. This education should involve not only the individuals themselves but also their family members and supervisors.

In Japan, a measure was taken to abolish national judo tournaments for elementary school students in 2022, due to concerns about excessive coaching of children, such as prioritizing victory (50). However, before this problem becomes more serious, another comprehensive action/policy is needed by the association to address the problem. In terms of Japanese junior high school girls, weight gain of about 3–4 kg per year is usually desirable (51). Therefore, for athletes, especially those in the lightweight to middleweight classes whose height growth was affected this time, to compete in the same class for three years in junior high school would mean that they are competing against normal growth. Children should eat according to their appetites without fear of weight gain. It is not in our budget to have a dietitian on every team in the country. However, nutrition education, which has been limited to top athletes, could be improved by creating new materials and disseminating information widely, or by incorporating lectures on the concept of weight loss and other topics into leadership training sessions. Another possible measure would be to require athletes to submit a plot of their growth curves at the time of competition.

This study has limitations. First, this study was based on data that relied on the recall of survey respondents, not actual measurements. Also, because it is a cross-sectional study, it is not possible to distinguish whether the exposures occurred before or after the observed results. Nonetheless, our study's strength lies in obtaining over 500 responses (Figure 1). However, the population was not randomly selected from the 3,667 registered female high school student athletes of the All Japan Judo Federation in 2022 (52) which is considered the population size, using a rigorous methodology.

## Data Availability

The raw data supporting the conclusions of this article will be made available by the authors, without undue reservation.
